# Anethole reduces oxidative stress and improves *in vitro* survival and activation of primordial follicles

**DOI:** 10.1590/1414-431X20187129

**Published:** 2018-05-28

**Authors:** N.A.R. Sá, J.B. Bruno, D.D. Guerreiro, J. Cadenas, B.G. Alves, F.W.S. Cibin, J.H. Leal-Cardoso, E.L. Gastal, J.R. Figueiredo

**Affiliations:** 1 Laboratório de Manipulação de Oócitos e Folículos Pré-antrais (LAMOFOPA), Faculdade de Medicina Veterinária, Universidade Estadual do Ceará, Fortaleza, CE, Brasil; 2 Laboratório de Biologia da Reprodução, Instituto de Ciências Biomédicas, Universidade Federal de Uberlândia, Uberlândia, MG, Brasil; 3 Laboratório de Biotecnologia da Reprodução (Biotech), Campus Uruguaiana, Universidade Federal do Pampa, Uruguaiana, RS, Brasil; 4 Laboratório de Eletrofisiologia (LEF), Instituto Superior de Ciências Biomédicas, Universidade Estadual do Ceará, Fortaleza, CE, Brasil; 5 Department of Animal Science, Food and Nutrition, Southern Illinois University, Carbondale, Illinois, USA

**Keywords:** Antioxidant, Anethole, ROS, Primordial follicle, Caprine

## Abstract

Primordial follicles, the main source of oocytes in the ovary, are essential for the maintenance of fertility throughout the reproductive lifespan. To the best of our knowledge, there are no reports describing the effect of anethole on this important ovarian follicle population. The aim of the study was to investigate the effect of different anethole concentrations on the *in vitro* culture of caprine preantral follicles enclosed in ovarian tissue. Randomized ovarian fragments were fixed immediately (non-cultured treatment) or distributed into five treatments: α-MEM^+^ (cultured control), α-MEM^+^ supplemented with ascorbic acid at 50 μg/mL (AA), and anethole at 30 (AN30), 300 (AN300), or 2000 µg/mL (AN2000), for 1 or 7 days. After 7 days of culture, a significantly higher percentage of morphologically normal follicles was observed when anethole at 2000 μg/mL was used. For both culture times, a greater percentage of growing follicles was observed with the AN30 treatment compared to AA and AN2000 treatments. Anethole at 30 and 2000 µg/mL concentrations at days 1 and 7 of culture resulted in significantly larger follicular diameter than in the cultured control treatment. Anethole at 30 µg/mL concentration at day 7 showed significantly greater oocyte diameter than the other treatments, except when compared to the AN2000 treatment. At day 7 of culture, levels of reactive oxygen species (ROS) were significantly lower in the AN30 treatment than the other treatments. In conclusion, supplementation of culture medium with anethole improves survival and early follicle development at different concentrations in the caprine species.

## Introduction

Primordial follicles are essential for the maintenance of fertility throughout the reproductive lives of mammalian females (1). Notwithstanding, the vast majority of this follicular category is lost due to the natural process named atresia. In order to recover and use these follicles, preantral follicle culture is an important tool to study *in vitro* folliculogenesis, including the effect of different substances (2).

Studies demonstrated that follicles cultured *in vitro* have lower developmental capability than those grown *in vivo* (3). Under *in vitro* conditions, follicles may be exposed to supra-physiological oxygen concentration (20%) (4). This may ultimately increase the production of reactive oxygen species (ROS), promoting oxidative stress (5).

Several studies have shown that high ROS levels produced during *in vitro* culture impair follicle development by damaging their cellular and molecular structures (for review, see Agarwal et al. (6). In this regard, a great variety of antioxidants such as selenium (7), α-tocopherol (8), ascorbic acid (5,9), and more recently, anethole (10) have been added to the culture media to reduce ROS production.

Anethole, the major constituent of the essential oil extracted from *Croton zehntneri Pax & K.Hoffm* (Euphorbiaceae), has been used in folk medicine as well as in the food and cosmetic industries (11,12). Hence, this compound may act as an anti-inflammatory (13), anesthetic (14), anticarcinogen (15), and antioxidant (10,13,15). Recently, anethole has been shown to improve the number of meiotically competent oocytes when added during *in vitro* culture of isolated caprine secondary follicles (10). Nevertheless, to the best of our knowledge, there are no reports describing the effect of anethole on *in vitro* culture of early stage preantral follicles (i.e., primordial, transition, and primary). The originality of the present study is based on the fact that follicular requirements vary according to the stage of follicle development. It has been shown recently that the transcriptional profiles of caprine secondary and early antral follicles differ in 2466 genes (16). Furthermore, Cadenas et al. (17) reported that caprine preantral and early antral follicles behave differently under the same culture conditions.

The objective of the present study was to evaluate the effect of different anethole concentrations on caprine *in vitro* early folliculogenesis. For this purpose, the following end points were evaluated: i) survival, ii) activation, iii) follicle and oocyte diameters, iv) cell proliferation, and v) ROS production of caprine early preantral follicles cultured *in vitro* enclosed in the ovarian tissue. In this study, caprine ovaries were used once the goat model had been reported to be appropriate for studying the effects of factors on *in vitro* folliculogenesis, with potential translational aspects for assisted reproductive techniques in human. Several similarities do exist between human and goat ovaries, such as size, organ texture, preantral follicle diameter, and folliculogenesis length (18).

## Material and Methods

### Chemicals and media

Unless otherwise mentioned, the culture media, ascorbic acid, anethole, and other chemicals used in the present experiment were purchased from Sigma-Aldrich (USA).

### Source of ovaries

All procedures and the research protocol (#1698730) were approved by the Ethics and Animal Use Committee (CEUA) of Universidade Estadual do Ceará (UECE), Brazil. Ovaries (n=10) from five adult crossbred goats (1–3 years old) were collected at a local slaughterhouse. The surrounding fat tissue and ligaments were removed and the ovaries were washed in 70% alcohol, followed by two washes in minimum essential medium (MEM) plus HEPES (MEM-HEPES). The ovaries were placed into tubes containing 15 mL of MEM-HEPES, supplemented with penicillin (100 μg/mL) and streptomycin (100 μg/mL) and then transported to the laboratory at 4°C within 1 h (19).

### Culture medium

The basic medium used was α-MEM^+^ (M5650, pH 7.2 - 7.4) supplemented with 1.25 mg/mL bovine serum albumin (BSA), ITS (10 μg/mL insulin, 5.5 μg/mL transferrin, 5 ng/mL selenium), 2 mM glutamine, and 2 mM hypoxanthine, which was also named α-MEM^+^. Incubation was carried out at 39°C in 5% CO_2_ in air for 1 or 7 days. Fresh media were prepared immediately before use and incubated for 1 h prior to use, with 1 mL in each well. The remaining fragments were individually cultured in a 24-well plate containing 1 mL α-MEM^+^ culture medium. The culture medium was replaced with fresh medium every 2 days. Each treatment was replicated five times.

### Experimental design

In the laboratory, the ovarian cortex of each pair (n=5) was removed with a sterile scalpel and divided into 22 fragments (3 × 3 × 1 mm). Two fragments were randomly taken and immediately fixed for the non-cultured control treatment as described below. The remaining fragments were randomly distributed (two fragments per treatment) into the five treatments as follows: α-MEM^+^ (cultured control), α-MEM^+^ supplemented with ascorbic acid at 50 μg/mL (AA), and anethole at 30 (AN30), 300 (AN300), or 2000 µg/mL (AN2000). The concentration of ascorbic acid (20), and anethole (30, 300, and 2000 µg/mL) were chosen based on previous studies (10,21).

### Evaluation of follicular morphology

Fresh, cultured control and treated cultured ovarian fragments were fixed in buffered 4% paraformaldehyde in phosphate buffered saline (PBS) for 12 h at 4°C, dehydrated in a graded series of ethanol, clarified with xylene, embedded in paraffin wax, and serially sectioned into 7 μm thickness. The sections were stained with periodic acid Schiff (PAS) and hematoxylin. For morphological evaluation, anonymous coded slides were examined on a microscope (Nikon, Japan) under 400× magnification, and the follicles were classified according to integrity and developmental stage.

The developmental stages of follicles have been defined previously (2) as: i) primordial (one layer of flattened granulosa cells around the oocyte); ii) transitional (one layer of flattened to cuboidal granulosa cells around the oocyte); iii) primary (a single layer of cuboidal granulosa cells); iv) secondary (two or more layers of cuboidal granulosa cells). The percentage of primordial and developing follicles was calculated on day 0 (control) and after 1 or 7 days of culture in each treatment. In addition, follicular and oocyte diameter were evaluated using only normal follicles on days 0, 1, and 7 of culture. Only follicles with a visible oocyte nucleus were evaluated to avoid counting the same follicle twice. Furthermore, follicles were classified as either morphologically normal (follicles containing an intact oocyte and granulosa cells well organized in layers without pyknotic nucleus) or degenerated (oocyte with a pycnotic nucleus, retracted cytoplasm, or disorganized granulosa cells detached from the basement membrane) (22).

### Immunohistochemical evaluation

**Figure 1.**
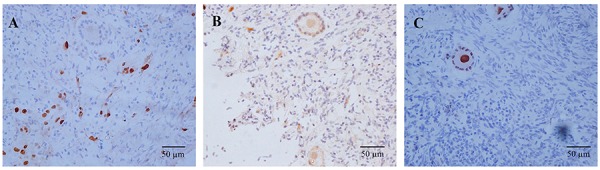
Distribution of PCNA immunoreactivity. A, absent, B, weak (<50%), or C, strong (>50%) in cells in the ovary by immunohistochemistry method. Bars=50 μm.

Ovarian tissue derived from the non-cultured control treatment, as well as from the cultured control, AA, AN30, AN300, and AN2000 treatments were evaluated by proliferating cell nuclear antigen (PCNA). The blockage for endogenous peroxidase activity, endogenous biotin, and nonspecific binding was performed by incubation in 5% normal goat serum and 3% triton x1000 diluted in PBS. After epitopes activation, slides were incubated overnight at 4°C with antiPCNA (1:300, AB2426, ABCAN, Inc., USA). Thereafter, the slides were washed in PBS and incubated with biotinylated anti-rabbit immunoglobulin G (IgG) secondary antibody (1:500, AB97049, ABCAN, Inc., USA). After that, the slides were washed, and the location of the protein was developed with diaminobenzidine (DAB; 1 drop of DAB for 1 mL of substratum, K346811-2; Dako, Inc., USA). Finally, the sections were counterstained with hematoxylin. The negative controls were carried out by replacing the primary antibody. Once mounted, the slides were visualized under a light microscope. In all treatments, primordial and developing follicles were evaluated. Follicles with at least one granulosa cell stained for PCNA were considered as positively stained. The scores attributed to the immunostaining ranged from weak to strong, according to the signal intensity indicated by the brown staining (Figure 1). The immunostaining was classified as absent (Figure 1A), weak (<50%, Figure 1B), or strong (>50%, Figure 1C) (23).

### Reactive oxygen species (ROS) levels

Levels of ROS were determined by a spectrofluorimetric method (24), using 2′,7′-dihydrodichlorofluorescein diacetate (DCHF-DA) assay. Sample aliquot (50 µL) was incubated at room temperature with 10 µL of DCHF-DA (1 mM). The oxidation of DCHF-DA to fluorescent dichlorofluorescein (DCF) was measured for the detection of intracellular ROS. The DCF fluorescence intensity emission was recorded at 520 nm (with 480 nm excitation) 2 h after the addition of DCHF-DA to the medium.

### Statistical analyses

Statistical analyses were carried out using Sigma Plot 11 (Systat Software Inc., USA). The percentage of morphologically normal follicles, follicular activation, and cell proliferation among treatments and days of culture were analyzed by the chi-squared test. Data without normally distribution (Shapiro-Wilk test) was transformed in base 10 logarithm. Comparisons of means (ROS, follicular, and oocyte diameters) were performed by Kruskal-Wallis (among treatments) and Mann-Whitney (between days of culture) tests. Data are reported as percentage and as means±SEM. Differences were considered to be significant when P<0.05.

## Results

### Follicle survival before and after culture

**Figure 2.**
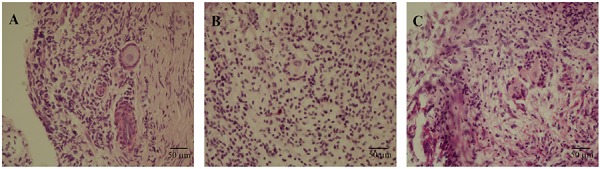
Morphologic aspects of preantral follicles after 7 days of culture in 30 µg/mL anethole treatment. *A*, normal follicle, *B* and *C*, abnormal follicles. Bars=50 µm.

A total of 4,589 preantral follicles were analyzed by classical histology. Normal (Figure 2A) and degenerated (Figure 2B and C) follicles after 7 days of culture in 30 μg/mL anethole treatment are shown.

The percentage of morphologically normal preantral follicles in the non-cultured control treatment and after 1 or 7 days of *in vitro* culture in all treatments is shown in Table 1. In all treatments, the percentage of normal follicles was lower (P<0.05) than in the non-cultured control treatment, and was reduced (P<0.05) from day 1 to day 7 of culture. At day 1 of culture, anethole at 30 and 300 µg/mL reduced (P<0.05) the percentage of normal follicles when compared to the cultured control treatment. However, when anethole at 2000 µg/mL was used, the greatest (P<0.05) percentage of normal follicles on day 7 was obtained.

**Table 1.** Percentage of morphologically normal preantral follicles in non-cultured ovarian tissue and in tissue cultured for 1 or 7 days in control medium with or without supplementation of 50 μg/mL of ascorbic acid (AA) or anethole 30 (AN30), 300 (AN300) or 2000 μg/mL (AN2000).

Treatments	Normal preantral follicles (%)		
	Day 0	Day 1	Day 7
Non-cultured control	92.3 (430/466)		
Cultured control		62.5 (364/582)*^aA^	36.3 (149/410)*^bA^
AA		60.2 (283/470)*^aAB^	36.6 (167/456)*^bA^
AN30		55.3 (273/494)*^aBC^	37.8 (84/222)*^bA^
AN300		52.2 (216/414)*^aC^	34.7 (87/251)*^bA^
AN2000		59.1 (319/540)*^aAB^	51.9 (181/349)*^bB^

*P<0.05 compared to non-cultured control group. ^a,b^Within days in the same treatment; ^A,B,C^Within treatments in the same period. Different superscript letters indicate significant difference (chi-squared test).

### Activation of primordial follicles after culture

The percentage of activation of preantral follicles in non-cultured control treatment and after 1 or 7 days of culture is shown in Table 2. As early as day 1 of culture, a reduction (P<0.05) in the percentage of primordial follicles with a concomitant increase (P<0.05) in the percentage of growing follicles was observed in all treatments compared to the non-cultured control treatment. In addition, in all treatments, the percentage of growing follicles increased (P<0.05) throughout the culture period. At day 1 of culture, AA and anethole supplementation increased (P<0.05) the percentage of growing follicles compared to the cultured control treatment. For both culture times, a higher (P<0.05) percentage of growing follicles was observed in the AN30 treatment compared to AA and AN2000 treatments.

**Table 2.** Percentage of primordial and growing follicles in non-cultured ovarian tissue and tissue cultured for 1 or 7 days in control medium with or without supplementation of 50 μg/mL of ascorbic acid (AA) or anethole at 30 (AN30), 300 (AN300) or 2000 μg/mL (AN2000).

Treatments	Primordial follicles (%)			Growing follicles (%)		
	Day 0	Day 1	Day 7	Day 0	Day 1	Day 7
Non-cultured control	58.8 (253/430)			41.16 (177/430)		
Cultured control		43.1 (157/364)*^aA^	6.0 (9/149)*^bAB^		56.9 (207/364)*^aA^	94.0 (140/149)*^bAB^
AA		31.8 (90/283)*^aB^	8.4 (14/167)*^bA^		68.2 (193/283)*^aB^	91.6 (153/167)*^bA^
AN30		24.2 (66/273)*^aC^	1.2 (1/84)*^bB^		75.8 (207/273)*^aC^	98.8 (83/84)*^bB^
AN300		26.4 (57/216)*^aBC^	2.3 (2/87)*^bB^		73.6 (159/216)*^aBC^	97.7 (85/87)*^bB^
AN2000		32.3 (103/319)*^aB^	8.8 (16/181)*^bA^		67.7 (216/319)*^aB^	91.2 (165/181)*^bA^

*P<0.05 compared to non-cultured control group. ^a,b^Within days in the same treatment; ^A,B,C^Within treatments in the same period. Different superscript letters indicate significant difference (chi-squared test).

### *In vitro* growth of preantral follicles

The follicular and oocyte diameters in the non-cultured control treatment and after 1 or 7 days of culture are shown in Table 3. Only anethole at 30 and 2000 µg/mL concentrations showed greater (P<0.05) follicular (days 1 and 7) and oocyte (day 7) diameters when compared to non-cultured control treatment. Anethole at 30 and 2000 µg/mL concentrations at days 1 and 7 of culture resulted in larger (P<0.05) follicular diameter than in the cultured control treatment; furthermore, both concentrations yielded larger (P<0.05) follicular diameter at day 7 compared to AA treatment. Anethole at 30 µg/mL concentration at day 7 showed greater (P<0.05) oocyte diameter than the other treatments, except when compared to AN2000 treatment.

**Table 3.** Follicle and oocyte diameters in non-cultured ovarian tissue and tissue cultured for 1 or 7 days in control medium with or without supplementation of 50 μg/mL of ascorbic acid (AA) or anethole at 30 (AN30), 300 (AN300) or 2000 μg/mL (AN2000).

Treatments	Follicles (µm)			Oocyte (µm)		
	Day 0	Day 1	Day 7	Day 0	Day 1	Day 7
Non-cultured control	24.6±0.5			18.8±0.3		
Cultured control		23.7±0.9^aA^	24.5±0.8^aA^		19.6±0.6^aAB^	17.7±0.7^aAC^
AA		25.0±1.1^aAB^	24.8±0.9^aA^		18.5±0.6^aA^	18.1±0.7^aAC^
AN30		27.0±1.0*^aB^	28.7±1.0*^aB^		20.3±0.7^aB^	21.1±0.8*^aB^
AN300		26.1±1.1^aAB^	24.8±0.7^aA^		19.1±0.6^aAB^	17.2±0.4*^bC^
AN2000		27.8±1.1*^aB^	27.3±0.9*^aB^		19.9±0.7^aAB^	19.5±0.7*^AB^

Data are reported as means±SE. *P<0.05 compared to non-cultured control group. ^a,b^Within days in the same treatment; ^A,B,C^Within treatments in the same period. Different superscript letters indicate significant difference. Kruskal-Wallis (among treatments) and Mann-Whitney (between days of culture) tests were used.

### ROS levels after culture

The levels of ROS in the culture medium at days 1 and 7 are shown in Table 4. At day 1 of culture, anethole at 30 and 2000 µg/mL reduced (P<0.05) the ROS levels compared to the cultured control treatment. In addition, AN2000 treatment showed lower (P<0.05) levels of ROS than AA treatment. However, at day 7 of culture, AN30 treatment showed the lowest (P<0.05) level of ROS. Furthermore, ROS production increased (P<0.05) from day 1 to day 7 of culture in all treatments, except in the cultured control and AN30 treatments.

**Table 4.** Levels of reactive oxygen species (ROS) measured in the culture medium of ovarian tissue cultured for 1 or 7 days in control medium with or without supplementation of 50 μg/mL of ascorbic acid (AA) or anethole at 30 (AN30), 300 (AN300) or 2000 μg/mL (AN2000).

Treatments	ROS	
	Day 1	Day 7
Cultured control	14.3±2.8^aA^	18.9±1.3^aA^
AA	11.1±1.7^aAC^	19.2±1.1^bA^
AN30	7.8±1.1^aBC^	11.7±1.6^aB^
AN300	8.5±1.0^aAB^	16.1±1.3^bA^
AN2000	7.1±0.9^aB^	20.8±2.9^bA^

Data are reported as means±SE. ^a,b^Within days in the same treatment. ^A,B,C^Within treatments in the same period. Different superscript letters indicate significant difference. Kruskal-Wallis (among treatments) and Mann-Whitney (between days of culture) tests were used.

### Detection of cell proliferation by PCNA marker

The percentage of labeling intensity for PCNA in preantral follicles in all treatments is shown in Table 5. A stronger (P<0.05) labeling intensity was found in granulosa cells of follicles in the AN30 treatment, compared to the non-cultured control, cultured control, and AA treatments.

**Table 5.** Percentage of labeling intensity for PCNA in preantral follicles in non-cultured ovarian tissue and tissue cultured for 7 days in control medium with or without supplementation of 50 μg/mL of ascorbic acid (AA) or anethole at 30 (AN30), 300 (AN300) or 2000 μg/mL (AN2000).

Treatments	Absent	Weak (<50%)	Strong (>50%)
Non-cultured control	41.2 (14/34)	35.3 (12/34)	23.5 (8/34)
Cultured control	31.6 (27/76)^A^	50.0 (38/76)^A^	18.4 (14/76)^A^
AA	32.2 (28/87)^A^	39.1 (34/87)^A^	28.7 (25/87)^AC^
AN30	21.3 (13/61)^A^	32.7 (20/61)^A^	46.0 (28/61)*^B^
AN300	19.1 (9/47)*^A^	44.7 (21/47)^A^	36.2 (17/47)^BC^
AN2000	19.1 (17/89)*^A^	38.2 (34/89)^A^	42.7 (38/89)^BC^

*P<0.05 compared to non-cultured control group. ^A,B,C^Within columns. Different superscript letters indicate significant difference (chi-squared test).

## Discussion

The present study investigated for the first time the effect of anethole on the *in vitro* culture of caprine preantral follicles enclosed in ovarian tissue. In general, anethole improved follicle survival and growth after 7 days of culture in different concentrations.

Anethole at the highest tested concentration (2000 μg/mL) had a greater percentage of morphologically normal follicles compared to the other treatments after culture. Anethole acts mainly as an antioxidant, as previously shown *in vivo* (25,26) and *in vitro* (10,13). Specifically, anethole increases intracellular levels of glutathione peroxidase and inhibits lipid peroxidation. Glutathione peroxidase can react with H_2_O_2_ and with a great variety of lipid hydroperoxides, and is considered responsible for protecting the cell membrane against oxidative damage (27). Therefore, the above potential mechanism may have been responsible for the improvement in follicular morphology maintenance observed in the present study. The addition of anethole at lower concentrations reduced the percentage of normal follicles compared to the cultured control treatment only at day 1 of culture. The anethole acts like antioxidants, by inhibiting lipid-peroxidation as well as hydroxyl radical scavengers. However, Chainy et al. (13) demonstrated that anethole in a lower concentration presented pro-oxidant action, which could explain the transient effect of anethole on the reduction of normal follicles on day 1 of culture.

*In vitro* culture conditions have a profound influence on follicular activation, i.e., the transition from primordial to developing follicles. In the present study, the addition of anethole to the control medium did not improve follicular activation after 7 days of culture. The follicular activation observed in the cultured control treatment might have been induced by the suitable composition of the culture medium used in this study, which is rich in nutrients, such as amino acids and carbohydrates (5). Previous reports in goats (28), cows (29), and baboons (30) have also not shown a medium-supplementation effect on *in vitro* primordial follicle activation.

Follicular and oocyte diameters after 7 days of *in vitro* culture were greater in the AN30 treatment than in the other treatments, except for the AN2000 treatment. It has previously been described that anethole stimulates granulosa cell proliferation, and consequently follicular growth, during *in vitro* culture of isolated goat secondary follicles (10). In addition, anethole has been involved in hepatic regeneration in mice (31). It has been shown that substances with antioxidant properties are directly related to a cascade of events that lead to growth and cell differentiation (32).

In the present study, ascorbic acid was used as an antioxidant positive control. The ascorbic acid addition did not reduce significantly ROS levels regardless the culture time. In general, antioxidant substances protect the cell from the ROS actions by different ways such as preventing, removing, and restoring these species (33). The essential action of the ascorbic acid is restoring the cell damages caused by ROS not necessarily by reducing ROS production. As a matter of fact, the results obtained in the present paper are in agreement with previous studies (10,33) that have shown that the ROS levels were not affected by ascorbic acid addition. On the other hand, anethole at 30 µg/mL reduced the ROS level at the end of the culture period compared to the other treatments and, interestingly, improved follicular and oocyte growth rates. The assessment of ROS production has been an important tool to determine the presence of free radicals that may exert a deleterious effect on cultured cells (34). In this sense, it has been demonstrated that an alteration of the optimal concentration of ROS initiates apoptosis, provoking damages in all follicular stages (10,34). An appropriate antioxidant concentration in the culture medium reduces the cell membrane impairment (35) due to its importance in maintaining cellular homeostasis, as well as in modulating physiological events that enhance cell development (36).

PCNA staining has commonly been used to evaluate cell proliferation, and has served as a marker for *in vitro* follicular development in several species such as human (37), mice (38), and caprine (32). In the present study, anethole at a dose of 30 µg/mL more effectively stimulated granulosa cell proliferation; furthermore, it also promoted greater follicular diameter. The positive PCNA staining observed in the present study might have been a result of DNA repairing (39) during the intense RNA transcription that occurs as the oocyte grows (40).

In conclusion, supplementation of culture medium with anethole improves survival and early follicle development in the caprine species. However, these parameters are affected differently by the concentration of anethole.
